# Prevalence of Human and Animal Fasciolosis in Butajira and Gilgel Gibe Health Demographic Surveillance System Sites in Ethiopia

**DOI:** 10.3390/tropicalmed8040208

**Published:** 2023-03-30

**Authors:** Samson Wakuma Abaya, Seid Tiku Mereta, Fikirte Demissie Tulu, Zeleke Mekonnen, Mio Ayana, Musse Girma, Hannah Rose Vineer, Siobhan M. Mor, Cyril Caminade, John Graham-Brown

**Affiliations:** 1 Department of Preventive Medicine, School of Public Health, College of Health Sciences, Addis Ababa University, Addis Ababa P.O. Box 9086, Ethiopia; 2 Department of Environmental Health Science and Technology, Jimma University, Jimma P.O. Box 378, Ethiopia; 3 School of Applied Natural Sciences, Adama Science and Technology University, Adama P.O. Box 1888, Ethiopia; 4 Department of Medical Laboratory Sciences, Institute of Health, Jimma University, Jimma P.O. Box 378, Ethiopia; 5 Akililu Lema Institute of Pathobiology Addis Ababa University, Addis Ababa P.O. Box 1176, Ethiopia; 6 Institute of Infection, Veterinary and Ecological Sciences, Leahurst Campus, University of Liverpool, Neston CH64 7TE, UK; 7 International Livestock Research Institute, Addis Ababa P.O. Box 5689, Ethiopia; 8 The Abdus Salam International Centre for Theoretical Physics (ICTP), Earth System Physics Department, Leonardo Building, Str. Costiera, 11, 34151 Trieste, Italy

**Keywords:** fasciolosis, Bio-X diagnostics, Health and Demographic Surveillance System, liver fluke, *Fasciola hepatica*

## Abstract

Fasciolosis is regarded as a major challenge to livestock productivity worldwide, but the burden of disease in humans has only started to receive some attention in the past three decades. The aim of this study was to determine the prevalence of human and animal fasciolosis and its determinant factors in the Gilgel Gibe and Butajira Health and Demographic Surveillance System (HDSS) sites in Ethiopia. A study was undertaken among 389 households across the two sites. Face-to-face interviews were conducted to investigate the knowledge, attitudes and practices of households with regard to fasciolosis. Stools from 377 children aged 7–15 years, and 775 animals (cattle, goats and sheep) were analyzed using a proprietary *Fasciola hepatica* (*F. hepatica*) coproantigen ELISA kit. The prevalence of fasciolosis in children was 0.5% and 1% in Butajira and Gilgel Gibe HDSS sites, respectively. The overall prevalence of animal fasciolosis was 29%, 29.2%, and 6% among cattle, sheep, and goats, respectively. More than half of the respondents from Gilgel Gibe (59%, n = 115) did not know that humans can be infected with *F. hepatica*. The majority of respondents in Gilgel Gibe (n = 124, 64%) and Butajira (n = 95, 50%) did not know the transmission route for fasciolosis. Grazing animals were 7 times more likely to be infected with fasciolosis than animals in cut-and-carry production systems (adjusted odds ratio [AOR] = 7.2; 95% confidence interval [CI]: 3.91–13.17). The findings indicated a lack of knowledge amongst local populations about fasciolosis. Thus, there is a need for public health awareness campaigns about fasciolosis in the study areas.

## 1. Introduction

Fasciolosis is a snail-borne parasitic disease that is caused by digenean trematodes of the genus *Fasciola* (“liver fluke”). This disease causes high morbidity and moderate to high mortality in many mammalian species, and is of particular importance in domestic ruminants (e.g., sheep and cattle). Estimates suggest that fasciolosis affects more than 300 million cattle and 250 million sheep worldwide [1]. The disease causes significant economic losses through mortality and reduced productivity (abattoir condemnations, reduced yields, etc.) [2]. Over the past three decades, fasciolosis has also gained more attention as an important (re-) emerging but neglected tropical disease in humans, predominantly affecting vulnerable people living in acute poverty [3]. Globally, 17 million people are estimated to be infected with liver fluke while about 180 million are at risk of infection [4]. Regions with high human endemic populations include Peru, Bolivia, and Ecuador in South America [5,6,7], as well as China, Turkey, Egypt, and Iran [8,9]. The distribution of fasciolosis is largely dependent on the presence of a competent intermediate snail intermediate host. *Fasciola hepatica* (intermediate host: *Galba truncatula*) is more commonly identified in temperate zones [10]. In contrast, *F. gigantica* (intermediate host: *Radix natalensis*) is an important cause of fasciolosis in the tropics and occurs throughout the western, sub-Saharan, and eastern Africa [11]. 

*Fasciola hepatica* is the most prevalent and important liver fluke in Ethiopia and is typically found in regions >1800 m above sea level, whilst *F. gigantica* is generally present <1200 m above sea level [12]. However, mixed infections with both *F. hepatica* and *F. gigant ica* are reported where the environment is conducive [13]. Studies have shown that animal fasciolosis occurs in almost all parts of Ethiopia [12,13,14,15,16,17]. Coprological and abattoir surveys conducted in WolaitaSodo town, Ethiopia, for example, indicated that the prevalence of bovine fasciolosis was about 72% [14]. Likewise, the prevalence of bovine fasciolosis in an abattoir survey in Jimma town, southwest Ethiopia, was about 47% [15]. A higher prevalence was recorded in the northern cities of Gondar (91%; abattoir survey) and in Bahir Dar (60%; coprological testing) [16]. Few studies have reported the general prevalence of the disease in humans in Ethiopia [18,19,20], although previous investigations have determined the prevalence of fasciolosis among school-aged children (who are considered the highest risk demographic) ranges from 2.5% to 9.8% [18,20].

Infection with *Fasciola* spp. can be confirmed using various diagnostic techniques, such as post-mortem, faecal egg detection, immunoassays (e.g., antibody ELISA), and copro-antigen ELISA. Each diagnostic technique has advantages and disadvantages: Post-mortem (PM) can facilitate definitive diagnosis as well as the degree of parasitic burden, stage, and severity of disease. Whilst PM is considered the “gold standard” for diagnosis in livestock, in abattoirs it is likely to have lower sensitivity and specificity due to high throughput and misidentifications [21].

Stool/faecal egg detection is suitable for diagnosis in both humans and animals; however, these methods have limitations [4]: Diagnosis can only be made after 8 to 10 weeks since eggs are produced only by patent adult infections [22]. Sensitivity is generally considered low compared to alternative diagnostic tests, with variation reported between egg count methods (Sedimentation techniques, kato-katz etc.), and species [23,24]. Egg count specificity is considered very high (100%), except following treatment when samples can remain positive for days to weeks [25].

Serological analysis using ELISA and other immunoassays are used in both livestock and human testing. These typically have a higher sensitivity than egg detection and enable diagnosis of pre-patent infection [26]. However, it is important to note that serum antibody ELISAs indicate exposure to infection; Serum antibody responses can remain elevated for several months post-treatment and, therefore, may also indicate historical infection. Possible serum cross-reactivity with antibodies raised against other parasitic infections may also be an issue [27,28].

A further alternative diagnostic method is the *F. hepatica* copro-antigen ELISA test [29]. This has a high sensitivity when compared with faecal egg detection, importantly including the detection of prepatent infection [30]. Furthermore, since copro-antigen ELISA involves direct antigen detection using a monoclonal antibody targeting *F. hepatica* excretory-secretory antigens (MM3), the same test protocol can be employed for all species (unlike serum antibody ELISAs which require species-specific detection antibodies). The copro-antigen ELISA has been assessed and validated for use in multiple species, including domestic livestock and humans [23,30,31,32]. 

Previous studies in Ethiopia assessed the prevalence of fasciolosis in humans and animals using rapid sedimentation technique for the detection of fluke eggs and abattoir surveys, although these methods have limitations of their own as previously discussed. In addition, previous studies on human and animal fasciolosis in Ethiopia have been conducted separately (compartmental disciplinary approach) and did not investigate both human and animal fasciolosis burden, and their link with the environment. Recent studies from other endemic areas have promoted the use of a One Health approach (i.e., human-animal-environment continuum) for efficient management and control of fasciolosis in humans and animals [33,34]. Therefore, this study aimed to determine the prevalence of human and animal fasciolosis and its social and environmental factors in two Health and Demographic Surveillance System (HDSS) sites in Ethiopia concurrently.

## 2. Methods

### 2.1. Study Sites

**Figure 1:**
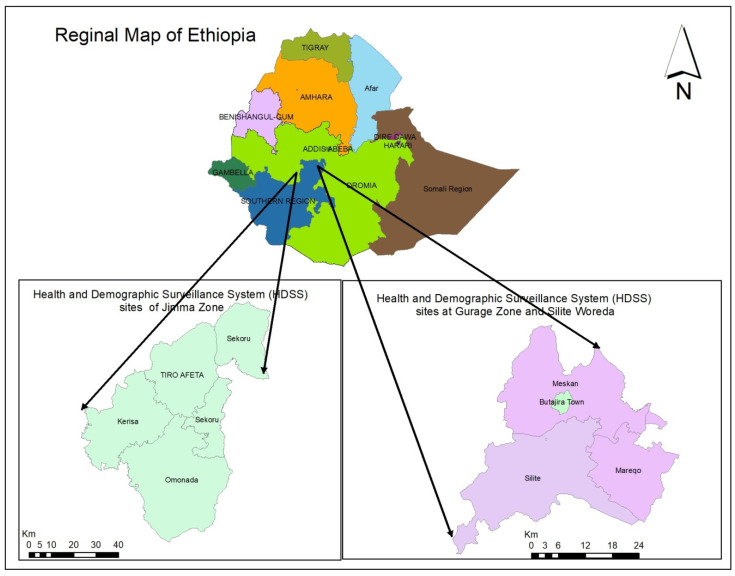
Map showing the location of the sites within Ethiopia. The Gilgil Gibe HDSS (**left**) is located in the Jimma zone of the Oromia region, whereas the Butajira HDSS (**right**) is located in the Gurage zone and Silite woreda of the Southern Nation and Nationality Region (SNNPR).

This study was conducted in Butajira in the Southern Nation and Nationality Region (SNNPR) and Gilgel Gibe in the Oromia Region of Ethiopia (Figure 1). Both sites are engaged in long-term continuous collection of health and demographic data. They thus provide timely and up-to-date community level data that are used by planners and policymakers to support evidence-based decision making and interventions. 

Butajira Rural Health Program—Addis Ababa University: The Butajira HDSS was established in 1987 and comprises of ten “kebeles” (lowest administrative unit in Ethiopia)—one urban and nine rural. The site is located 135 km southwest of the capital city, Addis Ababa. The site lies at an average of 2100 m with a range of 1750 to 3400 m above sea level. Annual rainfall varies between 900 and 1400 mm. The main rainy season occurs from June to September, with “small rains” commonly observed in March and April. The total population of the site was estimated at 88,642 in 2021. 

Gilgel Gibe Field Research Center (GGFRC)—Jimma University: The Gilgil Gibe HDSS was established in 2005 and comprises of eleven kebeles (eight rural and three urban). It borders the Gilgel Gibe hydroelectric dam within a 10 km radius. The center is located about 260 km southwest of Addis Ababa and 55 km northeast of Jimma town. Its agro-climatic zone is classified as midland. The total population of this region was estimated to be 69,972 in 2021.

### 2.2. Study Population

The study population included households in Butajira and Gilgil Gibe HDSS. Participants included adults aged >18 years (survey respondents), as well as children aged 7–15, and livestock (cattle and sheep/goats) in the same household.

Households were eligible to participate if they were located in one of the HDSS sites (Butajira or Gilgil Gibe), had at least one child aged 7–15 years, and owned at least one cow and one sheep/goat. Households that lived in the area for less than six months and respondents with serious illness and mental health problems were excluded from the study. For the questionnaire interview, only respondents older than 18 years old were included. Questionnaire respondents were either the children’s parents or primary carers.

### 2.3. Sample Size and Sampling Design

Sample size was estimated for each site and species (human, cattle, and small ruminants) using the single population proportion formula based on published estimates of fasciolosis prevalence (4% in humans and 15% in livestock based on a study carried out in north-western Ethiopia) [16,20]. Assuming a 95% confidence level and desired precision of 0.03 and 0.05 for human and animals, respectively, minimum sample sizes of 164 humans, 196 cattle, and 196 small ruminants (specifically, sheep and goats) were calculated for each site. Since the calculated sample size for livestock was larger than that for humans (196 vs. 164), we enrolled 196 households (i.e., 196 human samples, 196 cattle, and 196 small ruminant samples). Since we conducted the study in two ecologically diverse sites, the calculated sample size was multiplied by two, making the minimum total study sample to be 392 human stool samples and 784 animal faecal samples overall.

Multi-stage sampling was used to select households for the study. We estimated that one village would contain 30 households. Thus, seven villages were randomly selected per HDSS site. Subsequently, 196 households were randomly selected (without replacement) from the list of all households in the village for each HDSS site (Butajira and Gilgel Gibe). From the selected households, one child aged 7–15 was randomly selected for stool sample collection. Similarly, one cow and one small ruminant were randomly selected from each household for faecal sample collection.

### 2.4. Data Collection

Household visits and sampling were undertaken from January 2020 to May 2020.

Questionnaire: A questionnaire was developed based on a literature review [35,36] and contextualized to the local situation. The questionnaire was primarily used to collect information about socio-economics (age, sex, occupation, and education), water supply and handling, latrine facilities, animal husbandry (number and type of livestock) and behavioural factors (knowledge, attitudes, and practices). To ensure data collection quality, pre-testing was conducted prior to the main study data collection on 5% of the total household number (n = 20). Questions that were not easily understood by participants during this trial run were re-phrased. Main study data collection was then undertaken by four multi-disciplinary teams (one qualified veterinarian, one parasitologist, one environmental scientist, and one scientist experienced in survey data collection) at each HDSS site. Data collectors were trained over two days in the objectives of the study, questionnaire completion, and participant interview techniques. 

Sample collection: Sterile stool containers with screw caps were labelled and given to children with specific instructions on how to collect and submit their stool samples. Each sample was labelled with the participant identification number, and the child’s age, sex, date of collection, and site were recorded [30]. Voided faecal samples from livestock were collected from the night-time holding area of animals using sterile gloves to prevent cross contamination [37]. Samples were stored at 4 °C in sealed plastic bags and transported within 6 h of collection to the laboratory for subsequent processing, storage, and analysis. 

### 2.5. Laboratory Analysis

Faecal samples were analyzed as soon as possible following receipt at the Aklilu Lemma Institute of Pathobiology laboratory at Addis Ababa University (Butajira samples) and the Molecular laboratory at Jimma University (Gilgil Gibe samples). Faecal samples were analyzed using a commercially available *F. hepatica* copro-antigen ELISA kit according to manufacturer’s recommendations (BIO k 201/2; Bio-X diagnostics, Rochefort, France) [38]. Freshly collected cattle, sheep, goat, and human faeces were diluted in the dilution buffer (2 g + 2 mL for bovine and 0.5 g + 2 mL for ovine, caprine, and human samples) [31,39]. The diluted faeces suspension was then centrifuged at 1000× *g* for 5 min to separate the supernatant either for use with the ELISA, or short-term storage at −20 °C. Aliquots of each diluted sample supernatant were added to two micro wells (100 µL per well) sensitized with either *F. hepatica*-specific or non-specific polyclonal antibodies. Plates were then covered and incubated for 2 h at room temperature, after which the micro wells were rinsed with wash solution and drained twice. The biotin-conjugated anti-*F. hepatica* monoclonal detection antibody was then added (100 µL per well), incubated for 1 h at room temperature (21 °C ± 3 °C), and plate washing repeated. Subsequently, avidin-peroxidase was added (100 µL per well) and incubated for 1 h at room temperature (21 °C ± 3 °C), and plate washing repeated. Finally, chromogen was added to each test and control well (100 µL per well), and incubated in the dark at room temperature (21 °C ± 3 °C) for 10 min to allow colour development (enzymatic reaction), which was subsequently stopped by the addition of stopping solution (50 µL per well) of the kit. Optical density of the samples and positive controls were then read at 450 nm using the ELISA reader and the test results were calculated. Senior laboratory experts were involved in performing and overseeing immunoassays. All methods were cross-checked, both within the research team and the kit manufacturer (Bio-X diagnostics), prior to analysis.

### 2.6. Data Management and Data Analysis

The list of unique participant identifiers as well as the final data were kept confidential and accessed only by the research team. Data was manually entered into EpiData 3.1 then imported into SPSS (version 22) for data analysis. Missing values and outliers were checked carefully using frequency tabulation and residual plotting, and managed accordingly. Data were analyzed using descriptive statistics (mean, percentage, and standard deviation). The Pearson chi-square test was used to test the difference between the two HDSS sites for categorical variables.

Subsequently, logistic regression models were used to identify factors associated with fasciolosis. In the models, fasciolosis status was used as the dependent variable and socio-economic (i.e., age, sex, occupation, education, number of livestock, type, and number of herd); behavioural factors (i.e., knowledge, attitude, and practices) and animal husbandry practices were used as independent variables. Only those variables with *p*-value < 0.2 in the univariable logistic analyses were considered for inclusion in multivariable analysis. Explanatory variables with *p*-value ≤ 0.05 in the final multivariable model were considered statistically significant.

### 2.7. Ethical Considerations 

Ethical committees at the College of Health Sciences of Addis Ababa University (015/20/SPH), Jimma University Institute of Health (IRB000203/20), and University of Liverpool (7913) approved this study. Informed consent was obtained orally from each participant. Permission to sample household animals was also obtained. Participation in the study was voluntary and participants were free to withdraw participation at any time. Children returning a positive diagnosis for fasciolosis were referred to nearby health facilities.

## 3. Results

### 3.1. Sociodemographic Characteristics

A total of 389 households from Butajira (n = 194) and Gilgel Gibe (n = 195) participated in this study. Three households declined to participate, resulting in a response rate of 99%. Socio-demographic characteristics of respondents are summarised in Table 1. The majority of the participants who completed the questionnaire were female (53.5%, n = 208). About 53% (n = 207) of the respondents were aged between 31 to 45 years. More than half of the participants (56%, n = 218) could not read or write. The majority of participants were farmers that practiced both arable and livestock farming and lived in rural areas. Regarding income, the majority of households (56.3%, n = 219) earned more than 10,000 ETB (~$189 USD) per year.

Annual household income, age, and residence duration in the area were significantly different between the two HDSS sites, with a *p*-value of 0.001, 0.004, and 0.03 respectively.

### 3.2. Prevalence of Fasciolosis

The results of faecal testing are summarised for each species in Table 2. The prevalence of fasciolosis among children was 1 (0.5%) and 2 (1%) children in the Butajira and Gilgel Gibe HDSS sites, respectively. There was no significant difference in the prevalence of fasciolosis among children in the Gilgel Gibe and Butajira HDSS sites (*p*-value 0.60). Overall, the prevalence of fasciolosis in livestock was 26.7%. The highest prevalence of animal fasciolosis was found in Gilgel Gibe among cattle (97/195, 49.7%). 

The prevalence of fasciolosis in cattle and sheep was significantly higher in Gilgel Gibe than in Butajira HDSS sites, each with a *p*-value < 0.001.

### 3.3. Environmental Characteristics of Households 

The environmental characteristics of the households are shown in Table 3. Most households in Butajira (n = 154; 79.4%) and Gilgel Gibe (n = 139; 71.3%) had access to improved water sources (i.e., water source protected from outside contamination, and from faecal matter in particular). About 60% households (n = 54) in Butajira and 16% (n = 320) in Gilgel Gibe shared water sources with livestock. Households that grew vegetables were significantly higher in the Butajira than in the Gilgel Gibe HDSS sites (*p*-value < 0.001). All households in Gilgel Gibe, and more than 70% of households in Butajira, washed vegetables with untreated water. More than 93% of the households in both sites did not treat water obtained from sources other than pipe water. Over 86% of the households in both sites had latrines. More than 40% of households in both sites did not have effective/adequate hand-washing facilities. The majority of the households without latrines mentioned shortage of resources as a reason for not having latrines. 

No difference was found between the HDSS sites regarding access to an improved water source, sharing water with livestock, treating drinking water, availability of latrines, and availability of hand washing facilities (Table 3). 

### 3.4. Knowledge, Attitudes and Practices Related to Fasciolosis in Respondents

About 85% (n = 165) and 29% (n = 56) of respondents in Butajira and Gilgel Gibe had knowledge of fasciolosis, respectively (Table 4). However, less than 15% of the respondents in both sites had knowledge about the cause of fasciolosis. The majority of respondents from Gilgel Gibe (n = 115, 59%) did not know that humans can be infected by *Fasciola* spp. More than half of the respondents in both sites did not know the transmission route for fasciolosis. A majority of respondents in Butajira (n = 101, 52.1%) and Gilgel Gibe (n = 133, 68.2%) believed that fasciolosis can be prevented. Similarly, a majority of respondents from both sites thought that fasciolosis can be treated. About 25% (n = 46) of respondents in Butajira and 76% (n = 148) of respondents in Gilgel Gibe reported that they regularly eat raw vegetables. Most vegetable consumers in Gilgel Gibe consumed vegetables once a week. The most commonly consumed vegetable was tomato, followed by lettuce.

There was a statistically significant difference between respondents in Butajira and Gilgel Gibe regarding knowledge about fasciolosis infection, prevention, and control, each with a *p*-value < 0.001, but no statistical difference was found regarding the cause of fasciolosis (*p*-value 0.64). Raw vegetable consumption was significantly higher among households in Gilgel Gibe than in Butajira (*p*-value < 0.001) (Table 4).

### 3.5. Livestock Husbandry

More than 75% of respondents in both sites had separate shelters for ruminants (Table 5). Grazing was the only source of feed (100%) in the Gilgel Gibe site. A majority of respondents in Butajira (n = 192, 99%) and Gilgel Gibe (n = 176, 90%) clean the shelter every day. About 92% of respondents in Butajira and 82% in Gilgel Gibe de-worm their cattle (including albendazole and/or triclabendazole). A majority of the respondents in Butajira (n = 123, 63.4%) and Gilgel Gibe (n = 133, 68.2%) use animal dung to fertilize pastures. 

There was a significant difference regarding deworming cattle, frequency of deworming, and frequency of cleaning animal shelters between the Butajira and Gilgel Gibe HDSS sites, each with a *p*-value < 0.001, but there was no significant difference regarding separate shelters for ruminants and using animal dung as fertilizer between the two HDSS sites, with a *p*-value 0.65 and *p*-value 0.32, respectively. 

### 3.6. Factors Associated with Fasciolosis in Animals 

The results of multivariable analysis are shown in Table 6. After adjusting for other factors, grazing animals were 7 times more likely to be infected with *Fasciola* than animals fed with a cut-and-carry system (adjusted odds ratio [AOR] = 7.2, 95% confidence interval [CI]; 3.91–13.17). Furthermore, animals owned by farmers that had never used an anthelmintic to de-worm their cattle had twice the odds of being infected with *Fasciola* compared to animals owned by farmers that did practice de-worming (AOR = 2.2, 95% CI; 1.25–3.78). Finally, animals owned by people that used manure as a fertilizer had 1.7 times the odds of being infected with *Fasciola* compared to animals owned by farmers who did not use manure for fertilizer (AOR = 1.7, 95% CI; 1.16–2.56). 

## 4. Discussion

This study is the first to apply *F. hepatica* coproantigen ELISA to both human and livestock samples in Ethiopia. In this study, stool testing for *Fasciola* focused on children due to their status as a demographic with high risk of infection; former studies conducted in Bolivia, Peru, and Egypt indicated that most fasciolosis infections are found in school-aged children [40]. Furthermore, previous studies conducted in Tanzania and Ethiopia found a high prevalence of fasciolosis among school-aged children [20].

The average prevalence of fasciolosis infection among children for both study sites was 0.8%, which is comparable with an estimate for Chile (0.7%) [10]. However, the present finding was lower than the previous studies conducted among school children in Ethiopia, where prevalence ranged from 2.5% to 9.8% in Amhara Regional State [18], and 3.3% in Lake Tana Basin, north-west Ethiopia [20]. These differences may be due to several factors—the first being differences in ecological setting. Previous studies indicate that environmental conditions, such as temperature, rainfall, and soil moisture, play important roles on the development of the snail intermediate host [41,42]. These previous study sites in Ethiopia had several permanent water bodies, marshy areas, and rivers that could lead to higher prevalence rates in livestock and, therefore, people. Secondly, in this study, the majority of respondents had access to improved water sources and latrines compared with limited access to improved sanitation in previous investigations [18]. 

The overall mean prevalence of animal fasciolosis infection was 27%, with a higher prevalence recorded in the Gilgel Gibe site compared to Butajira (46.7% vs. 8.3%; *p* < 0.001). This difference could be related to extensive water resource development in Gilgel Gibe, which includes dam development for hydroelectric power. This potentially creates favourable conditions for the snail intermediate hosts and, ultimately, for the transmission of fasciolosis.

The prevalence of bovine fasciolosis In this study was similar with previous estimates for different parts of Ethiopia: Haramaya (24.4%) [43], Mekelle area (24.3%) [44], and Kombolcha (28%) [45], but lower than a study conducted in the northeast Amhara Region (47.1%) [24] and eight other administrative regions of Ethiopia (61%) [46]. These differences could be due to differences in sample size, environmental factors, and diagnosis methods as previously discussed.

In this study, the prevalence of fasciolosis in sheep was much higher than in goats. This finding is consistent with an abattoir survey conducted in central Ethiopia where prevalence was 20.75% in sheep and 1.59% in goats [47]. This difference might be related to differences in the eating behaviours of these two species; sheep are grazers while goats are browsers, thus sheep are more exposed to metacercaria as it is attached to the grass [48,49]. This difference is also likely to have implications for the epidemiological role each small ruminant species plays in maintenance and transmission of fasciolosis from a One Health perspective.

The multivariable analysis in this study indicated that grazing animals had a higher odds ratio of infection with *Fasciola* than animals fed on cut-and-carry systems. This finding is in agreement with a study conducted in Nepal among buffalo that indicated that hay making and cut-and-carry systems reduced the risk of *F. gigantica* infections [50]. Similarly, other studies have reported that cattle fed via grazing are more likely to have a *Fasciola* spp. infection as compared to those fed on silage [51]. This is because the silage production facilitates the destruction of metacercaria that are usually attached to the grass [52].

The present study also indicated that de-worming was associated with a reduced risk of infection with *Fasciola*. This is in agreement with a study conducted by Nguyen et al. who reported a higher prevalence of fasciolosis among cattle without anthelmintic treatment in Vietnam [53]. In this study, common flukicides included albendazole and triclabendazole. There is little literature on the susceptibility of fluke to these therapeutics in Ethiopia, however, given the association found here between use of anthelmintics and *F. hepatica* infection status, we conclude these are likely to be effective.

This study found that using dung as fertilizer was more likely to cause fasciolosis. This finding is similar to a study conducted by Kurnianto et al. who reported that animals on farms with poor manure management practices were more likely to have fasciolosis [54]. Using manure as fertilizer can increase contamination of pasture or paddy fields with *Fasciola* eggs, and increase the likelihood of local transmission to competent intermediate hosts.

Although a low prevalence rate of fasciolosis was found in children in this study, fasciolosis should still be considered as a public health risk; there was a high prevalence rate among animals living in the area capable of contributing to local transmission in the local aquatic environments, thereby increasing human disease risk. In combination with an apparent lack of awareness and knowledge of these local populations regarding risk factors associated with fasciolosis. In Butajira, 15% of respondents did not know about the disease—while in Gilgel Gibe, 70% did not know what fasciolosis was. Consequently, any changes likely to increase *F. hepatica* transmission potential in these regions, including those previously discussed in association with a higher prevalence in animals in Gilgel Gibe, would not be mitigated by public awareness and effective preventative measures. Furthermore, due to a low prevalence in humans, fasciolosis may be low on the list of clinical differentials, and therefore missed in pursuit of treatments of alternative pathology (e.g., neoplasia).

One of the limitations of this study was the cross-sectional design, implemented at a single time-point that doesn’t consider the seasonal variations in infection rates.

## 5. Conclusions

The prevalence of human fasciolosis was low in Butajira and Gilgel Gibe HDSS; however, the prevalence of animal fasciolosis was high. Feeding type, lack of de-worming of cattle, and use of animal dung for fertilization were risk factors significantly associated with fasciolosis in animals. The findings in this study also indicated the disparities in the level of knowledge across the two sites about fasciolosis. Thus, for proper prevention and control, there is a need for public health awareness campaigns about fasciolosis in these regions.

## Figures and Tables

**Table 1:** Socio-demographic characteristics of questionnaire respondents in a study of fasciolosis in Butajira and Gilgel Gibe HDSS sites, Ethiopia.

Characteristic		HDSS Site			Significance Level (Butajira versus Gilgel Gibe HDSS Site)
		Butajira n = 194 (%)	Gilgel Gibe n = 195 (%)	Both Sites n = 389 (%)	
Sex	Male	90 (46.4)	91 (46.7)	181 (46.5)	0.96 ^†^
	Female	104 (53.6)	104 (53.3)	208 (53.5)	
Age (years)	18–30	38 (19.6)	66 (33.8)	104 (26.7)	0.004 ^†^
	31–45	118 (60.8)	89 (45.6)	207 (53.2)	
	46–55	20 (10.3)	27 (13.8)	47 (12.1)	
	>55	18 (9.3)	13 (6.7)	31 (8.0)	
Educational status	Can’t read or write	99 (51.0)	119 (61.0)	218 (56.0)	0.14 ^†^
	Primary Education	83 (42.8)	67 (34.4)	150 (38.6)	
	Secondary education & above	12 (6.2)	9 (4.6)	21 (5.4)	
Family size	≤5 people	35 (18.0)	28 (14.4)	63 (16.2)	0.32 ^†^
	>5 people	159 (82.0)	167 (85.6)	326 (83.8)	
Residence	Urban	1 (0.5)	-	1 (0.3)	N
	Rural	193 (99.5)	195 (100)	388 (99.7)	
Residence duration in the area	≤5 years	2 (1.0)	9 (4.6)	11 (2.8)	0.03 ^†^
	>5 years	192 (99.0)	186 (95.4)	378 (97.2)	
Occupation	Farmer	95 (49.0)	110 (56.4)	205 (52.7)	0.11 ^†^
	Housewife	92 (47.4)	83 (42.6)	175 (45.0)	
	Government & private employee	7 (3.6)	2(1.0)	9 (2.3)	
		1 (0.5)	2 (1.0)	3 (0.8)	
Annual household income	<5000 ETB (~$96 USD)	75 (38.7)	5 (2.6)	80 (20.6)	<0.001 ^†^
	5000–ETB (~$96–189 USD)	60 (30.9)	30 (15.3)	90 (23.1)	
	>ETB (~$189 USD)	59 (30.4)	160 (82.1)	219 (56.3)	

n: number of study participants; (%) percentage; ^†^ Pearson chi-square test; N: not calculated, owing to low numbers.

**Table 2:** Number of cases and prevalence of fasciolosis among humans and animals in the Butajira and Gilgel Gibe HDSS sites, Ethiopia.

Species	HDSS Sites									
	Butajira			Gilgel Gibe				Both Sites		
	No. Sampled	Faecal Antigen Test Results	n (%)	No. Sampled	Faecal Antigen Test Results	n (%)	Significance Level (Butajira versus Gilgel Gibe HDSS Sites)	No. Sampled	Faecal Antigen Test Results	n (%)
Children	182	Positive	1 (0.5)	195	Positive	2 (1.0)	0.60 ^†^	377	Positive	3 (0.8)
		Negative	181 (99.5)		Negative	193 (99.0)			Negative	374 (99.2)
Cattle	194	Positive	16 (8.2)	195	Positive	97 (49.7)	<0.001 ^†^	389	Positive	113 (29.0)
		Negative	178 (91.8)		Negative	98 (50.3)			Negative	276 (71.0)
Sheep	141	Positive	13 (9.2)	195	Positive	85 (43.6)	<0.001 ^†^	336	Positive	98 (29.2)
		Negative	128 (90.8)		Negative	110 (56.4)			Negative	238 (70.8)
Goat	50	Positive	3 (6.0)	-	Positive	-	N	50	Positive	3 (6.0)
		Negative	47 (94.0)		Negative	-			Negative	47 (94.0)

n: frequency; (%) percentage; ^†^ Pearson chi-square test; N: not calculated, owing to missing numbers.

**Table 3:** Environmental characteristics of households in a study of fasciolosis in the Butajira and Gilgel Gibe HDSS sites, Ethiopia.

Variable		HDSS Site		*p*-Value
		Butajira n (%)	Gilgel Gibe n (%)	
Source of water for drinking		n = 194	n = 195	
	Protected water source	154 (79.4)	139 (71.3)	0.06 ^†^
	Unprotected water source	40 (21.6)	56 (28.7)	
Sharing water source with livestock		*n = 194*	*n = 195*	
	Yes	53 (27.3)	32 (16.4)	0.09 ^†^
	No	141 (72.7)	163 (83.6)	
Do you grow vegetables?		n = 194	n = 195	
	Yes	89 (45.9)	37 (19.0)	<0.001 ^†^
	No	105 (54.1)	158 (81.0)	
Source of water for vegetable growth		*n = 89*	*n = 37*	
	Surface water	53 (59.6)	34 (91.9)	<0.001 ^†^
	Groundwater	36 (40.4)	3 (8.1)	
Type of water for washing vegetable		*n = 89*	*n = 37*	
	Treated water	24 (27.0)	-	N
	Untreated water	65 (73.0)	37 (100)	
Do you treat drinking water that comes from sources other than piped water?		n = 194	n = 195	
	Yes	13 (6.7)	12 (6.2)	0.83 ^†^
	No	181 (93.3)	183 (93.8)	
Water treatment methods		*n = 13*	*n = 12*	
	Chlorination	-	1 (8.3)	N
	Boiling	10 (76.9)	9 (75.1)	
	Filtering	3(23.1)	1 (8.3)	
	Other	-	1 (8.3)	
Household latrine availability		n = 194	n = 195	
	Yes	168 (86.6)	174 (89.2)	0.43 ^†^
	No	26 (13.4)	21 (10.8)	
Type of latrine		*n = 168*	*n = 174*	
	Traditional latrine	168 (100%)	174 (100%)	N
Is there a hand-washing facility with soap around the latrine?		*n = 168*	*n = 174*	
	Yes, with soap	3 (1.8)	101 (58.0)	0.14 ^†^
	Yes, without soap	88 (52.4)	-	
	No facility	77 (45.8)	73 (42.0)	
If no toilet, what do you use?		*n = 26*	*n = 21*	
	Open field	19 (73.1)	21 (100)	N
	Share with neighborhood	7 (26.9)	-	
Reason for not having a latrine		*n = 26*	*n = 21*	
	Lack of space	1 (3.8)	-	N
	The water table is high	1 (3.8)	-	
	Soil type is not appropriate	7 (27.0)	-	
	Shortage of resources	16 (61.6)	21 (100)	
	Lack of awareness	1 (3.8)		

n: frequency; (%) percentage; ^†^ Pearson chi-square test; N: not calculated, owing to missing numbers.

**Table 4:** Knowledge, attitudes, and practices related to fasciolosis of participants in the Butajira and Gilgel Gibe HDSS sites, Ethiopia.

Variable		HDSS Site		*p*-Value
		Butajira n (%)	Gilgel Gibe n (%)	
Do you know a disease called liver fluke?				
	Yes	165 (85.1)	56 (28.7)	<0.001 ^†^
	No	29 (14.9)	139 (71.3)	
Do you know the cause of fasciolosis?				
	Yes	28 (14.4)	25 (12.8)	0.64 ^†^
	No	166 (85.6)	170 (87.2)	
What causes fasciolosis?		*n = 28*	*n = 25*	
	Bacterial infection	3 (10.7)	1 (4.0)	N
	Snails	-	1 (4.0)	
	Parasite worm	16 (57.1)	11 (44.0)	
	Don’t known/Not sure	9 (32.2)	12 (48.0)	
Can human be infected with *Fasciola* spp.?				
	Yes	162 (83.5)	80 (41.0)	<0.001 ^†^
	No	32 (16.5)	115 (59.0)	
What are the transmission routes of fasciolosis in humans?				
	Eating improperly washed vegetable	18 (9.3)	1 (0.5)	N
	Eating raw vegetables	21 (10.8)	1 (0.5)	
	Drinking impure water	27 (13.9)	-	
	Dirty kitchen utensils	6 (3.1)	1 (0.5)	
	Using contaminated water to irrigate crops	1 (0.5)	1 (0.5)	
	Eating raw meat/liver	71 (36.6)	71 (36.2)	
	I don’t know	95 (48.9)	124 (63.6)	
In your opinion can fasciolosis be prevented?		*n = 165*	*n = 56*	
	Yes	88 (53.3)	43 (76.8)	<0.001 ^†^
	No	77 (46.7)	13 (23.2)	
Do you think that human fasciolosis can be treated?		*n = 165*	*n = 56*	
	Yes	130 (78.8)	35 (62.5)	<0.001 ^†^
	No	15 (9.1)	6 (10.7)	
	Not known	20 (12.1)	15 (26.8)	
What should you do if you are infected with *Fasciola*?				
	Self-treatment	1 (0.5)	1 (0.5)	N
	Go traditional healer	179 (91.8)	-	
	Go to health institution	102 (52.6)	539 (91.8)	
	Others	15 (7.7)	45 (7.7)	
Do you eat raw vegetables?				
	Yes	46 (23.7)	148 (75.9)	<0.001 ^†^
	No	148 (76.3)	47 (24.1)	
Kind of vegetable consumed raw		*n = 46*	*n = 148*	
	Tomato	26 (56.5)	76 (51.4)	<0.001 ^†^
	Lettuce	16 (34.8)	71 (47.9)	
	Swiss chard	4 (8.7)	1 (0.7)	
If you notice in your meal any vegetables watered from surface water?				
	Not eat	57 (29.4)	185 (94.9)	N
	Eat when well cooked	136 (70.1)	4 (2.1)	
	Eat when treated carefully	-	4 (2.1)	
	Still eat raw	1 (0.5)	2 (1.0)	
How often do you eat raw vegetables?		*n = 46*	*n = 148*	
	Every day	-	3 (2.0)	N
	Once a week	33 (71.7)	101 (68.2)	
	Once a month	10 (21.7)	32 (21.6)	
	Once a year	2 (4.4)	3 (2.0)	
	Never	1 (2.2)	9 (6.2)	

n: frequency; (%) percentage; ^†^ Pearson chi-square test; N: not calculated, owing to missing numbers. Due to conditional logic, the total for individual questions does not always add up to the total number of respondents. Where this is the case, the denominator for individual questions is indicated in italics.

**Table 5:** Livestock husbandry practices of households in a study of fasciolosis in Butajira and Gilgel Gibe HDSS sites, Ethiopia.

Variable		HDSS Site, n (%)		*p*-Value
		Butajira n = 194	Gilgel Gibe n = 195	
Is there a separate shelter for ruminants				
	Yes	151 (77.8)	148 (75.9)	0.65 ^†^
	No	43 (22.2)	47 (24.1)	
Which form of animal husbandry do you practice?				
	Cut and carry	91 (46.9)	-	N
	Grazing	103 (53.1)	195 (100)	
How often do you clean the cattle shelter?				
	Every day	192 (99.0)	176 (90.3)	0.001 ^†^
	Every other day	1 (0.5)	7 (3.6)	
	Weekly	1 (0.5)	12 (6.2)	
What kind of water do you use to clean the shelter				
	Treated	34 (17.5)	-	N
	Untreated	160 (82.5)	195 (100)	
Have you ever dewormed your cattle?				
	Yes	187 (96.4)	160 (82.1)	<0.001 ^†^
	No	7 (3.6)	35 (17.9)	
How often do you deworm		*n = 187*	*n = 160*	
	Once a year	40 (21.4)	31 (19.4)	<0.001 ^†^
	Twice a year	57 (30.5)	97 (60.6)	
	More than twice a year	90 (48.1)	32 (20.0)	
Do you use animal dung to fertilizer pastures				
	Yes	123 (63.4)	133 (68.2)	0.32 ^†^
	No	71 (36.6)	62 (31.8)	

n: frequency; (%) percentage; ^†^ Pearson chi-square test; N: not calculated, owing to missing numbers. Due to conditional logic, the total for individual questions does not always add up to the total number of respondents. Where this is the case, the denominator for individual questions is indicated in italics.

**Table 6:** Factors associated with fasciolosis status in animals in the Butajira and Gilgel Gibe HDSS sites, Ethiopia.

Variable		Frequency	Fasciolosis Status		Univariable Analysis		Multivariable Analysis	
			Positive	Negative	OR (95% CI)	*p*-Value	AOR (95% CI)	*p*-Value
Is there a separate shelter for ruminants								
	Yes (reference)	595	161	434	1		1	
	No	180	53	127	1.1 (0.78–1.62)	0.5	1.0 (0.66–1.45)	0.9
Type of feed								
	Cut and carry (reference)	179	13	166	1		1	
	Grazing	596	201	395	6.5 (3.6–11.7)	0.001	7.2 (3.91–13.17)	0.001 *
How often do you clean the cattle shelter?								
	Every day (reference)	733	196	537	1		1	
	Every other day	16	7	9	2.1 (0.78–5.8)	0.13	1.3 (0.46–3.69)	0.68
	Weekly	26	11	15	2.0 (0.90–4.4)	0.08	1.4 (0.60–3.06)	0.46
Have you ever dewormed your cattle?								
	Yes (reference)	691	180	511	1		1	
	No	84	34	50	1.9 (1.3–3.1)	0.006	2.2 (1.25–3.78)	0.001 *
Do you use animal dung to fertilize pastures?								
	Yes	509	138	371	0.9 (0.66–1.29)	0.6	1.7 (1.16–2.56)	0.007 *
	No (reference)	266	76	190	1		1	

Asterisk denotes a statistically significant relationship (*p* < 0.05) between the explanatory and response variables. OR, odds ratio; AOR, adjusted odds ratio; CI, confidence interval.

## Data Availability

The dataset generated and/or analyzed during the present study is available from the corresponding author.
